# Biases arising from using linked administrative data for research: A conceptual framework from registration to analysis.

**DOI:** 10.23889/ijpds.v7i3.1800

**Published:** 2022-08-25

**Authors:** Richard Shaw, Katie Harron, Julia Pescarini, Elzo Júnior, Andressa Siroky, Desmond Campbell, Ruth Dundas, Maria Yury Ichihara, Mauricio Barreto, Vittal Katikireddi

**Affiliations:** 1 University of Glasgow; 2 UCL Great Ormond Street Institute of Child Health; 3 London School of Hygiene and Tropical Medicine; 4 Centre for Data and Knowledge Integration for Health (CIDACS)

## Objectives

Administrative data are primarily collected for operational processes and these processes can lead to sources of bias that may not be adequately considered by researchers. We provide a framework to help understand how biases might arise from using linked administrative data, and hopefully aid future study designs.

## Approach

We developed the conceptual framework based on the team’s experiences with the 100 Million Brazilian Cohort (100MCohort) which contains records of more than 131 million people whose families applied for social assistance between 2001 and 2018, linked to other administrative data sources. We provide examples from the 100MCohort of where and how in the linkage process different forms of bias could arise. We make recommendations on how biases might be addressed using commonly available external data.

## Results

The conceptual framework covers the whole data generating process from people and events occurring in the population through to deriving variables for analysis. The framework comprises three distinct stages: 1) Recording and registration of events in administrative systems such as Brazil’s Mortality Information System (SIM) and the Hospital Information System (SIH); 2) Linkage of different data sources, for example using exact matching via the Social Identification Number (NIS) in Brazil’s CadÚnico database or linkage algorithms; 3) Cleaning and coding data used both for analysis and linkage. The biases arising from linkage can be better understood by applying theory and making additional metadata available.

## Conclusion

Maximising the potential of administrative data for research requires a better understanding of how biases arise. This is best achieved by considering the entire data generating process, and better communication among all those involved in the data collection and linkage processes.

